# Electrocardiographic and biochemical analysis of anthracycline induced cardiotoxicity in breast cancer patients from Southern Sri Lanka

**DOI:** 10.1186/s12885-023-10673-0

**Published:** 2023-03-04

**Authors:** Jayasinghe Arachchige Nirosha Sandamali, Ruwani Punyakanthi Hewawasam, Madappuli Arachchige Chaminda Sri Sampath Fernando, Kamani Ayoma Perera Wijewardana Jayatilaka

**Affiliations:** 1 Department of Medical Laboratory Science, Faculty of Allied Health Sciences, University of Ruhuna, Galle, Sri Lanka; 2 Department of Biochemistry, Faculty of Medicine, University of Ruhuna, Galle, Sri Lanka; 3 Department of Economics & Statistics, Faculty of Social Sciences & Languages, Sabaragamuwa University of Sri Lanka, Belihuloya, Sri Lanka

**Keywords:** Anthracycline, Breast cancer patients, Cardiotoxicity, Electrocardiography, NT-proBNP, Troponin I

## Abstract

**Background:**

The clinical application of anthracycline chemotherapy is hindered due to the cumulative dose-dependent cardiotoxicity followed by the oxidative stress initiated during the mechanism of action of anthracyclines. Due to a lack of prevalence data regarding anthracycline-induced cardiotoxicity in Sri Lanka, this study was conducted to determine the prevalence of cardiotoxicity among breast cancer patients in Southern Sri Lanka in terms of electrocardiographic and cardiac biomarker investigations.

**Methods:**

A cross-sectional study with longitudinal follow-up was conducted among 196 cancer patients at the Teaching Hospital, Karapitiya, Sri Lanka to determine the incidence of acute and early-onset chronic cardiotoxicity. Data on electrocardiography and cardiac biomarkers were collected from each patient, one day before anthracycline (doxorubicin and epirubicin) chemotherapy, one day after the first dose, one day and six months after the last dose of anthracycline chemotherapy.

**Results:**

Prevalence of sub-clinical anthracycline-induced cardiotoxicity six months after the completion of anthracycline chemotherapy was significantly higher (*p* < 0.05) and there were strong, significant (*p* < 0.05) associations among echocardiography, electrocardiography measurements and cardiac biomarkers including troponin I and N-terminal pro-brain natriuretic peptides. The cumulative anthracycline dose, > 350 mg/m^2^ was the most significant risk factor associated with the sub-clinical cardiotoxicity in breast cancer patients under study.

**Conclusion:**

Since these results confirmed the unavoidable cardiotoxic changes following anthracycline chemotherapy, it is recommended to carry out long-term follow-ups in all patients who were treated with anthracycline therapy to increase their quality of life as cancer survivors.

## Background

Anthracyclines are a group of antibiotics that were isolated from *Streptomyces peucetius*, a species of actinobacteria and the introduction of them has dramatically improved the outlook of many cancers over the past 15 years [1]. Doxorubicin is the most commonly used and most effective chemotherapeutic agent in the anthracycline family [2]. However, the clinical use of anthracyclines is compromised by their toxic effects on healthy tissues [3]. Production of hydroxyl free radicals during the metabolism of anthracyclines leads to multi-organ toxicity and the myocardial tissues are more susceptible for the free radical-induced damage compared to the other tissues [4–6]. According to previous studies, the level of doxorubicin-induced cardiotoxicity is ten times greater than the toxicities associated with other tissues such as the liver, kidney, spleen and mitochondria are the most extensively and progressively damaged subcellular organelles in doxorubicin-induced cardiotoxicity [7, 8]. One reason for this may be due to the high-affinity binding of doxorubicin with cardiolipin, a phospholipid in the inner mitochondrial membrane, which plays an important role in mitochondrial function as it is required for the proper functioning of the proteins in the electron-transport chain [9]. Another reason for the vulnerability of the heart tissue to doxorubicin-induced cardiotoxicity is due to the presence of antioxidant enzymes such as peroxidase, catalase and superoxide dismutase at comparatively lower levels [10].

Since oxidative stress is the major mechanism involved in doxorubicin-induced cardiotoxicity, it is mediated through the accumulation of reactive oxygen species (ROS) in the intracellular spaces which are generated through redox cycling in mitochondria [11]. In addition to the redox cycling, ROS are also produced in the myocardium by activation of pro-oxidant enzymes including NADPH oxidase and xanthine oxidases. Since the balance between ROS production and antioxidant defense mechanism is more susceptible to disruption in the myocardial tissues, it induces deleterious effects in the heart tissues including DNA damage, cell death, mitochondrial dysfunction, disruption in calcium homeostasis, and defects in protein synthesis [12].

According to previous studies, cardiotoxicity induced by anthracyclines has one or more signs and symptoms including a reduction in the left ventricular ejection fraction (LVEF), signs associated with heart failures such as S3 gallop, tachycardia or both, reduction in LVEF from baseline that is in the range of less than or equal to 5% to less than 55% accompanying signs or symptoms of heart failure, or a reduction in LVEF in the range of equal to or greater than 10% to less than 55%, without accompanying signs or symptoms of heart failure [13]. Doxorubicin-induced cardiotoxicity can be acute or chronic [14]. Acute cardiotoxicity is usually evident during and within 2–3 days of administration of anthracyclines and the incidence is about 11%. The incidence of chronic cardiotoxicity is about 1.7% and it is primarily related to the cumulative dose of doxorubicin administered. Although there is no limit for the standard cumulative dose associated with cardiotoxicity induced by doxorubicin, the incidence of cardiovascular diseases increases with increased cumulative dose and an inter-individual variation was also reported [14, 15]. The incidence was about 3–5% when the cumulative dose was 400 mg/m^2^, 5–8% when the cumulative dose was 450 mg/m^2^, and 6–20% when the dose was 500 mg/ m^2^ [15].

When considering the prevalence of anthracycline-induced cardiotoxicity, only a few studies have been conducted in Asian countries and only one study was previously conducted in Sri Lanka and published by us based on only the echocardiographic parameters. Therefore, this study was conducted to determine the prevalence of anthracycline-induced cardiotoxicity in breast cancer patients admitted to the oncology unit, Teaching Hospital, Karapitiya, Sri Lanka based on electrocardiography (ECG) findings and cardiac biomarkers including cardiac troponin I (cTnI) and N terminal pro-brain natriuretic peptide (NT-pro BNP) since these parameters provide early identification of acute changes in the heart although the echocardiography requires some extent of damage to the heart for the diagnosis of cardiotoxicity [16].

## Methods

### Experimental procedure

A cross-sectional study with longitudinal follow-up was conducted following the declaration of Helsinki at the Teaching Hospital, Karapitiya, Sri Lanka. Ethical approval for the study was obtained from the Ethical Review Committee, Faculty of Medicine, University of Ruhuna, Sri Lanka (23.10.2014:3.10).

A-priori sample size calculator for multiple regression was used to determine the required sample size. The required sample size was calculated as 195 with an anticipated effect size (0.10), desired statistical power level (0.80), number of predictors (14), and probability level (0.05). However, it was expected to recruit a few additional patients to secure 195 observations after data cleaning. Initially, 205 patients were recruited to the study and ultimately 196 patients remained after excluding the dropouts.

Written informed consent was obtained from every patient before the data collection. The purpose of the study was clearly explained to each and every participant before obtaining the consent. Data was collected giving sufficient time and protecting privacy of the patients. Participants had the ability to reject the participation at any time after they were recruited to the study. An interviewer administered pretested questionnaire was used to collect the data. Anonymity and confidentiality of individual data were maintained throughout the study.

All newly diagnosed breast cancer patients above 18 years of age who were administered anthracycline and cyclophosphamide (AC) chemotherapy for the first time and who gave informed consent were enrolled in the study. Any patient who was in a critical state or end stage of cancer and who was unable to give informed consent, patients who received chemotherapy that do not belong to the anthracycline family, patients who were diagnosed with any structural heart disease or cardiomyopathy and patients who developed any acute non-cardiac complications were excluded from the study. In the hospital setting, anthracycline is administered only as a combination therapy, and only this combination was selected to limit the effect of other cardiotoxic chemotherapy agents. Once consent was given, an interviewer-administered questionnaire was used to collect the socio-demographic data and other relevant information related to the study.

Records of ECG reports and 5.0 mL of venous blood were collected from each patient who was administered with AC schedule as the first line treatment on the following four occasions; one day before chemotherapy (baseline), one day after the first dose of chemotherapy, one day after the last dose of chemotherapy and six months after the completion of chemotherapy. However, after the last dose of chemotherapy, most of the patients were undergone some therapeutics considered the second part of the adjuvant program including taxane-trastuzumab and chest wall irradiation which can affect the results obtained six months after the completion of anthracycline chemotherapy.

**Fig. 1:**
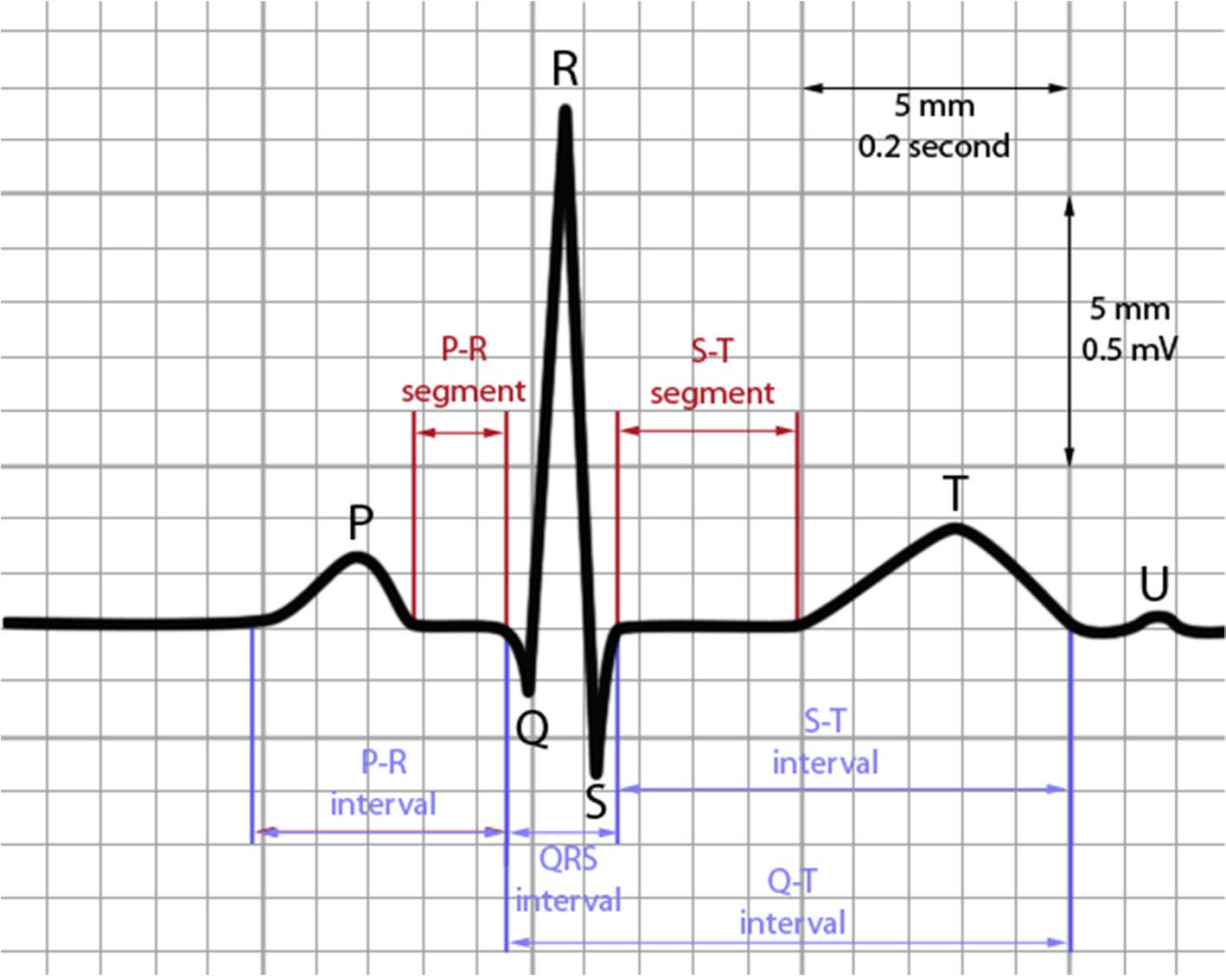
Sample of standard ECG paper which shows the scale of voltage in vertical axis and time in the horizontal axis. (*Extracted from *http://www.medicine.mcgill.ca/physio/vlab/cardio/introecg.htm*)*

PR interval, QRS duration, and QTc interval were calculated from ECG records to investigate the cardiotoxic events in patients treated with anthracycline chemotherapy as changes were reported in these parameters in previous studies [13, 16–18]. The following guidelines were used when calculating these parameters [19]. The standard ECG paper has a grid of small and large squares and in the horizontal axis, each small square indicates 40 ms (ms) in time. PR interval was calculated from the beginning of the P wave to the first deflection of the QRS complex as shown in Fig. 1. QRS duration was obtained from the first deflection of the QRS complex to the end of the QRS complex at the isoelectric line and the QT interval was calculated from the first deflection of the QRS complex to the end of the T wave at the isoelectric line. The QTc interval was calculated from the Fridericia formula; QTc = QT / RR ^1/3^ (RR; heart rate) with the help of the QTc calculator (https://www.mdcalc.com/).

Venous blood (5.0 mL) was collected into a plain tube for the estimation of NT-proBNP and cTnI concentration. The serum was separated and stored at -85° C until the ELISA tests were performed. The concentration of cTnI was estimated according to the method of Fromm [20], and the concentration of NT-proBNP was estimated according to the method of Bay et al. [21].

### Statistical analysis

The statistical analysis was performed using MINITAB 18 software package. This study was planned with 14 predictor variables. It was expected to use the multiple linear regression model as the main analytical tool. The demographic profile and other clinical characteristics of the patients enrolled in the study (age, weight, height, BMI, cumulative dose of anthracycline, radiation, systolic BP, and diastolic BP) were presented in the form of mean and standard deviation. Other baseline characteristics of the patients enrolled in the study included Nottingham grade of cancer, type anthracycline, number of anthracycline cycles, therapeutics administered as the second part of the adjuvant program, blood relatives with cancer, marital status, diabetes, dyslipidemia and ER, PR and Her-2 receptor status were presented as percentage frequencies.

### ECG analysis

One-way Analysis of Variance (One-way ANOVA) test was applied to test the hypothesis of equal means at 0.05 level of significance to analyze the ECG changes one day after the first dose of chemotherapy, one day after the last dose of chemotherapy, and six months after the completion of anthracycline chemotherapy in terms of the PR interval, QRS duration, and QTc interval. Then the Tukey’s multiple comparison test was applied to identify the different pairs of means. Pearson’s product-moment correlation coefficient was used to analyze the linear relationship between ECG changes and six continuous variables including age, weight, height, BMI, cumulative dose, and radiation. Chi-squared test was used to investigate the association between sub-clinical cardiotoxicity identified from echocardiographic findings [22] and ECG parameters. Binary logistic regression analysis was carried out to investigate the relationship between the cardiac risk factors and the deviation of ECG interval values compared to the normal intervals.

### Biochemical analysis

One way ANOVA was used to analyze the mean differences of biochemical results including cTnI and NT-proBNP at the four time points (baseline, one day after the first dose of chemotherapy, one day after the last dose of chemotherapy, and six months after the completion of chemotherapy). Tukey's multiple comparison test was applied to identify the different pairs of means. Pearson's product moment correlation coefficient was used to analyze the relationship between biochemical results six months after the completion of anthracycline chemotherapy and continuous variables including age, weight, height, BMI, cumulative dose of anthracycline, and chest wall irradiation. Chi-squared test was used to analyze the association between sub-clinical cardiotoxicity (EF difference > 10% in echocardiography) and biochemical changes as well as the association with ECG parameter changes. Binary logistic regression analysis was carried out to investigate the relationship between the cardiac risk factors and the changes in biochemical parameters. The odds ratios (ORs) and 95% confidence intervals (CIs) were estimated from regression coefficients. *P* < 0.05 was considered significant.

## Results

Patients selected for the study (196) had no clinical signs or symptoms of cardiac dysfunction during the study period. All patients were females with breast cancer as there were limitations in selecting patients who received only an anthracycline drug regimen in the actual clinical setup. In the Sri Lankan clinical setting, anthracyclines are always prescribed as a combination therapy and many combinations have other cardiotoxic agents and anthracycline and cyclophosphamide schedule (AC schedule) is the most commonly used regimen which includes anthracyclines. Therefore, in the present study, the cardiotoxicity of the patients who received anthracycline and cyclophosphamide schedule was evaluated. Anthracyclines at 60 mg/m^2^ and cyclophosphamide at 600 mg/m^2^ were prescribed for the breast cancer patients admitted to the Teaching Hospital, Karapitiya, Sri Lanka as the standard drug regimen.

**Fig. 2:**
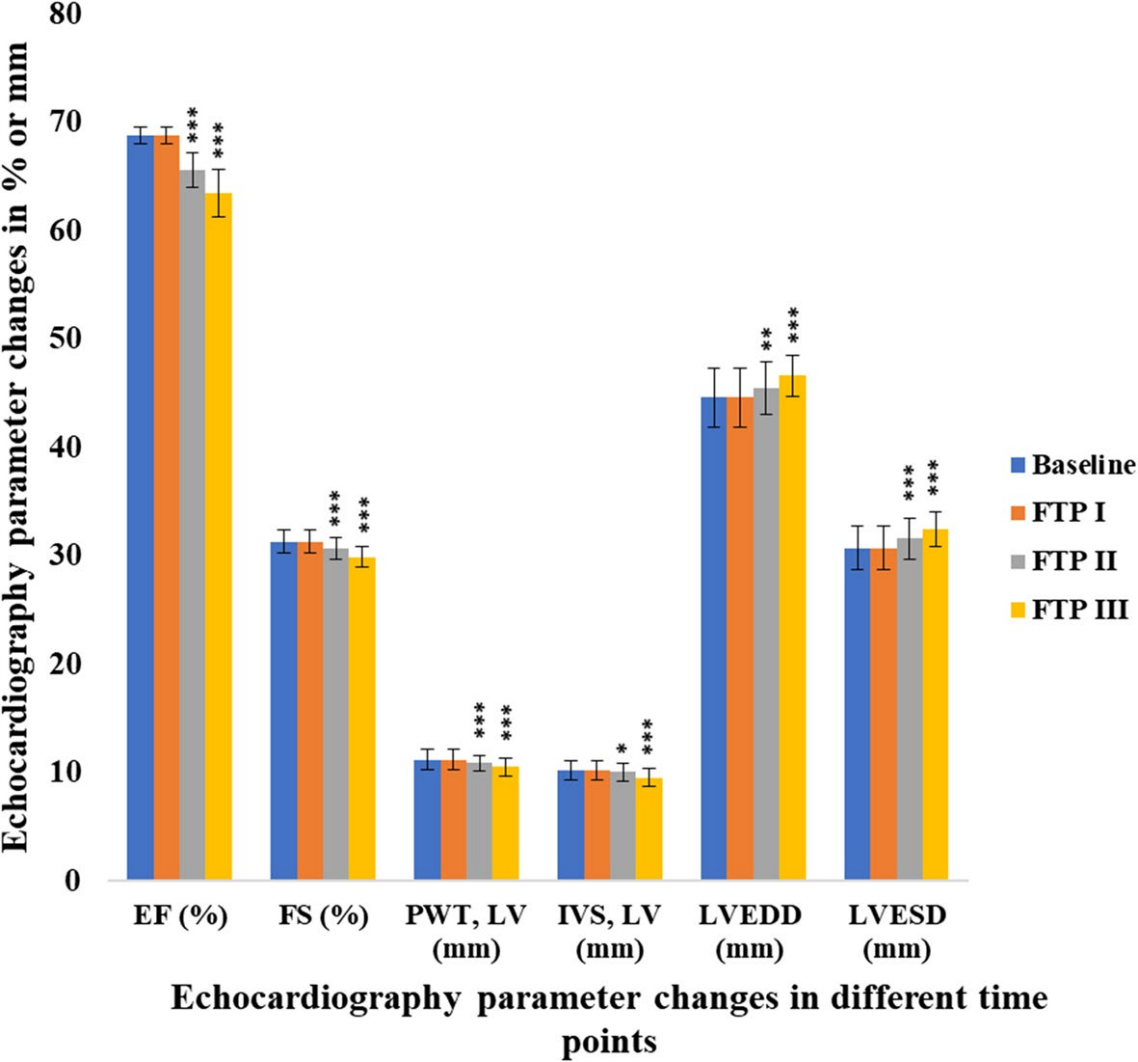
Comparison of echocardiographic parameters among the four time points. Baseline; One day prior to anthracycline chemotherapy, FTP I; One day after 1^st^ dose of anthracycline chemotherapy, FTP II; One day after last dose of anthracycline chemotherapy, FTP III; Six months after completion of anthracycline chemotherapy. All values are expressed as mean ± SD (*n* = 196). *p* values *˂ 0.05, **˂ 0.01, ***˂0.001 compared to the baseline readings. (Paired t-test was applied to compare echocardiographic parameters between the baseline and one day and six months after the completion of anthracycline chemotherapy). FTP; Follow up time point, EF; ejection fraction, FS; fractioning shortening, PWT, LV; posterior wall thickness (left ventricle), IVS, LV; thickness of inter-ventricular septum (left ventricle), LVEDD; left ventricular end diastolic diameter, LVESD; left ventricular end systolic diameter, SD; standard deviation

The demographic profile and other clinical characteristics of the patients enrolled in the study (age, weight, height, BMI, cumulative dose, radiation, systolic BP, and diastolic BP) are presented as mean ± standard deviation in Table 1 [22]. Other baseline characteristics of the patients enrolled in the study included Nottingham grade of cancer, type of anthracycline, number of anthracycline cycles, therapeutics administered as the second part of the adjuvant program, blood relatives with cancer, marital status, diabetes, dyslipidemia and ER, PR and Her-2 receptor status are also presented as percentage frequencies in Table 2 [22]. A summary of the echocardiographic analysis is presented in Fig. 2 [22].

**Table 1:** Demographic and clinical characteristics of the study participants

Demographic and clinical characteristics of patients	Mean ± SD
Age (years)	53.6 ± 11.0
Weight (kg)	53.5 ± 10.3
Height (m)	1.5 ± 0.1
BMI (kg/m^2^)	24.2 ± 4.3
Cumulative dose (mg/m^2^)	340.8 ± 37.0
Radiation received (Gy)	49.4 ± 22.9
Systolic BP (mmHg)	115.3 ± 8.1
Diastolic BP (mmHg)	74.9 ± 5.8

*BMI* Body Mass Index, *BP* Blood pressure

**Table 2:** Baseline characteristics of the study participants (*n* = 196)

Nottingham grade of cancer		Receptor status	
Low nuclear grade in situ CA	01 (0.5%)	ER positives	50 (25.5%)
Grade I	30 (15.3%)	PR positives	38 (19.4%)
Grade II	101 (51.5%)	Her 2 positives	02 (1.0%)
Grade III	64 (32.7%)	ER & PR positives	94 (48.0%)
**Type of anthracycline**		ER & Her 2 positives	06 (3.1%)
Doxorubicin	157 (80.1%)	PR & Her 2 positives	03 (1.5%)
Epirubicin	39 (19.9%)	ER, PR & Her 2 positives	03 (1.5%)
**Number of anthracycline cycles given**		**Other therapeutics used as the second part of the adjuvant programme**	
04 cycles	193 (98.5%)	None	05 (2.6%)
06 cycles	03 (1.5%)	Docetaxel	128 (65.3%)
**Patients with**		Paclitaxel	46 (23.4%)
Diabetes mellitus	84 (42.9%)	Nano Paclitaxel	17 (8.7%)
Dyslipidaemia	69 (35.2%)	**Blood relatives**	
Neither 43 (21.9%)		With cancer	47 (24.0%)
**Marital status**		Without cancer	149 (76.0%)
Married	148 (75.5%)		
Unmarried	48 (24.5%)		

*CA* carcinoma, *ER* estrogen receptor, *PR* progesterone receptor, *Her 2* human epidermal growth factor receptor 2

### ECG analysis

The mean PR interval, QRS duration, and QTc interval of patients one day before initiation of chemotherapy (baseline value) were 141.22 ± 18.30 ms, 99.6 ± 16.45 ms, and, 434.6 ± 9.72 ms respectively as shown in Table 3. According to the Tukey simultaneous tests for differences of means, there was a significant difference (*p* < 0.001) between the baseline values of ECG parameters including QRS duration and QTc interval, and the ECG readings one day after the last dose of chemotherapy and six months after the completion of anthracycline chemotherapy. Although, there is no significant difference in PR interval between baseline values and one day after the first dose of chemotherapy and one day after the last dose of chemotherapy, a significant (*p*˂0.001) difference was observed six months after the completion of chemotherapy. The normal value for PR interval in females is ˂200 ms, QRS duration is ˂120 ms and QTc interval is ˂440 ms [19, 23]. In the study group, 5.6% of patients had a QRS duration of ˃120 ms one day after the first dose of chemotherapy and 17.9% and 21.4% of patients had a QRS duration of ˃120 ms one day after the last dose of chemotherapy, and six months after the completion of chemotherapy respectively. There were 14.8% of patients one day after the first dose, 30.1% one day after the last dose, and 31.6% six months after the completion of anthracycline chemotherapy who had QTc interval ˃440 ms. Although some patients had increased PR interval one day after and six months after the completion of anthracycline chemotherapy, none of them had a PR interval of ˃200 ms.

**Table 3:** Comparison of electrocardiographic (ECG) parameters at four time points

ECG parameters Mean (95%CI)	One day prior to anthracycline chemotherapy (baseline)	One day after 1^st^ dose of anthracycline chemotherapy (FTP I)		One day after last dose of anthracycline chemotherapy (FTP II)	Six months after completion of anthracycline chemotherapy (FTP III)	
PR interval (ms)	141.6 (139.0, 144.3)	141.6 (139.0, 144.3)		143.3 (140.6, 146.0)	149.5 (146.8, 152.2)	
QRS duration (ms)	99.6 (96.5, 102.7)	103.8 (100.7, 106.9)		110.4 (107.3, 113.5)	113.3 (110.2, 116.4)	
QTc interval (ms)	434.6 (432.1, 437.1)	438.7 (436.2, 441.2)		445.0 (442.5, 447.5)	448.0 (445.5, 450.5)	
Mean difference (95% CI)	FTP I—Baseline	FTP II—Baseline	FTP III—Baseline	FTP II—FTP I	FTP III—FTP I	FTP III—FTP II
PR interval (ms)	0.0 (-4.9, 4.9)	1.6 (-3.3, 6.6)	7.9*** (2.9, 12.8)	1.6 (-3.3, 6.6)	7.9*** (2.9, 12.8)	6.2** (1.3, 11.2)
QRS duration (ms)	4.2 (-1.5, 9.9)	10.8*** (5.1, 16.5)	13.7*** (8.0, 19.4)	6.6* (0.9, 12.4)	9.5*** (3.8, 15.2)	2.9 (-2.9, 8.6)
QTc interval (ms)	4.1 (-0.5, 8.7)	10.4*** (5.8, 15.0)	13.4*** (8.7, 18.0)	6.3** (1.7, 10.9)	9.3*** (4.7, 13.9)	3.0 (-1.7, 7.6)

All values are expressed as mean (95% CI) (*n* = 196). One-way Analysis of Variance (One-way ANOVA) test was applied to test the hypothesis of equal means at 0.05 level of significance. A p-vale of 0.000 + (*p* values *< 0.05, **< 0.01, ***< 0.001) indicated at least two different means. Then the Tukey’s multiple comparison test was applied to identify the different pairs of means)

*ms* milli seconds, *CI* confidence interval, *FTP* Follow up time point

FTP I; One day after 1st dose of anthracycline chemotherapy, FTP II; One day after last dose of anthracycline chemotherapy, FTP III; Six months after completion of anthracycline chemotherapy

**Fig. 3:**
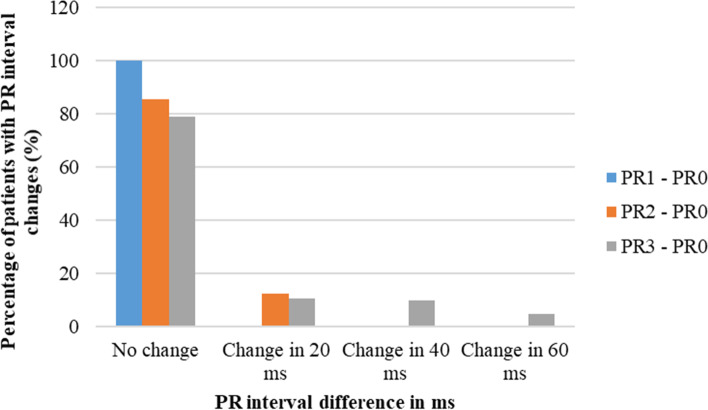
Percentage of patients with PR interval changes in milli seconds compared to the baseline PR interval. PR0; PR interval reading at the baseline. PR1; PR interval reading one day after the first dose of anthracycline chemotherapy (FTP I). PR2; PR interval reading one day after the last dose of anthracycline chemotherapy (FTP II). PR3; PR interval reading six months after the completion of anthracycline chemotherapy (FTP III). FTP; Follow up time point

**Fig. 4:**
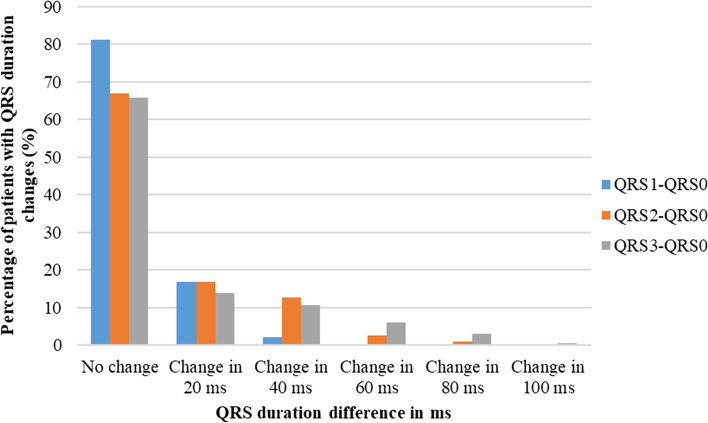
Percentage of patients with QRS duration changes in milli seconds compared to the baseline QRS duration. QRS0; QRS duration reading at the baseline. QRS1; QRS duration reading one day after the first dose of anthracycline chemotherapy (FTP I). QRS2; QRS duration reading one day after the last dose of anthracycline chemotherapy (FTP II). QRS3; QRS duration reading six months after the completion of anthracycline chemotherapy (FTP III). FTP; Follow up time point

**Fig. 5:**
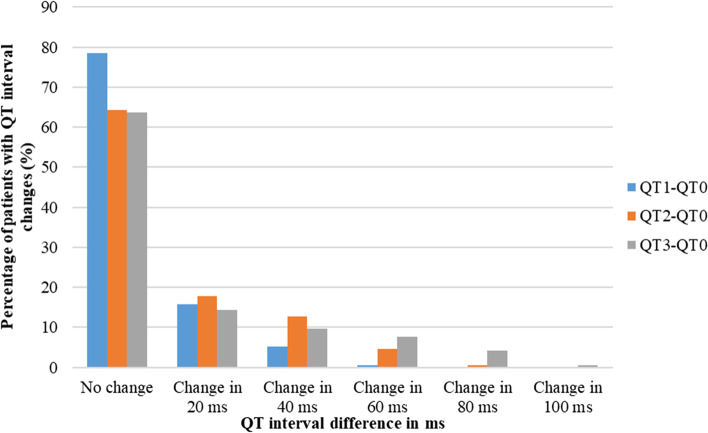
Percentage of patients with QTc interval changes in milli seconds compared to the baseline QTc interval. QT0; QTc interval reading at the baseline. QT1; QTc interval reading one day after the first dose of anthracycline chemotherapy (FTP I). QT2; QTc interval reading one day after the last dose of anthracycline chemotherapy (FTP II). QT3; QTc interval reading six months after the completion of anthracycline chemotherapy (FTP III)

Changes in PR interval, QRS duration, and QTc interval compared to the baseline values are shown in Figs. 3, 4 and 5. All the patients recruited to the study had no change in PR interval one day after the first dose of anthracycline chemotherapy. Changes in PR interval gradually increased one day and six months after the completion of chemotherapy. About 81% of patients and 79% of patients had no changes in QRS duration and QTc interval respectively one day after the first dose of chemotherapy. However, the percentage of patients who had changes in QRS duration and QTc interval increased with time. QRS change in 20 ms was relatively high one day after the first dose of anthracycline chemotherapy and the highest changes in QRS duration including 60 ms, 80 ms, and 100 ms were observed six months after the completion of chemotherapy. When considering the QTc interval changes, more than 63% of patients had no changes in QTc interval even six months after the completion of anthracycline chemotherapy. The changes in QTc interval in 20 ms were mostly observed one day after the last dose of chemotherapy and the same as the other parameters the highest changes were observed six months after the completion of chemotherapy.

As the highest percentage of patients with a QRS duration of ˃120 ms and QTc interval of ˃440 ms was observed six months after the completion of anthracycline chemotherapy, the relationship between these two parameters and the continuous variables including age, weight, height, BMI, cumulative dose of anthracycline and chest wall irradiation was investigated and results are shown in Table 4. Both ECG parameters had a weak positive linear relationship with age and the cumulative dose of anthracyclines.

**Table 4:** Correlation between the basic characteristics and the changes in electrocardiographic (ECG) parameters six months after the completion of anthracycline chemotherapy

Variables	Correlation coefficient (*p* value)	
	Difference in QRS duration	Difference in QTc interval
Age	0.40 (0.001)**	0.38 (0.001)**
Weight	0.08 (0.252)	0.07 (0.340)
Height	0.05 (0.453)	0.06 (0.447)
BMI	0.07 (0.355)	0.05 (0.479)
Cumulative dose of anthracycline	0.36 (0.001)**	0.32 (0.001)**
Radiation	0.01 (0.877)	0.06 (0.425)

*BMI* Body Mass Index

*p* values *< 0.05, **< 0.01, ***< 0.001 considered as significant

The Chi-squared test was performed to investigate whether the ECG parameters including QRS duration and QTc interval beyond the reference range six months after the completion of anthracycline chemotherapy is correlated to the sub-clinical cardiotoxic findings of echocardiography (patients with EF difference ≥ 10% after the six months of completion of anthracycline chemotherapy) and the results are shown in Tables 5 and 6. According to the results it was observed that there is a strong relationship between changes in EF values in echocardiographic measures and changes in ECG parameters.

**Table 5:** Association between sub-clinical cardiotoxicity (EF changes ≥ 10%) and the changes in electrocardiographic (ECG) parameters six months after the completion of anthracycline chemotherapy

	QRS duration ≤ 120 ms	QRS duration > 120 ms
EF difference (EF_3_-EF_0_) < 10%	113 (85.6%)	19 (14.4%)
EF difference (EF_3_-EF_0_) ≥ 10%	41 (65.1%)	23 (34.9%)

*n* = 196, *p*-value = 0.001**

*EF* Ejection fraction

*p* values *< 0.05, **< 0.01, ***< 0.001 considered as significant

**Table 6:** Association between sub-clinical cardiotoxicity (EF changes ≥ 10%) and the changes in electrocardiographic (ECG) parameters six months after the completion of anthracycline chemotherapy

	QTc interval ≤ 440 ms	QTc interval > 440 ms
EF difference (EF_3_-EF_0_) < 10%	100 (75.8%)	32 (24.2%)
EF difference (EF_3_-EF_0_) ≥ 10%	34 (53.1%)	30 (46.9%)

*n* = 196, *p*-value = 0.002**

EF; Ejection fraction, *p* values *< 0.05, **< 0.01, ***< 0.001 considered as significant

The association of risk factors for cardiomyopathy with the deviation of ECG parameters, QRS duration, and QTc interval from the baseline value six months after the completion of anthracycline chemotherapy was investigated using binary logistic regression and results are shown in Table 7. Age of more than 60 years was found to be the most significantly contributed risk factor for both ECG parameters, QRS duration, and QTc interval which had an OR of 2.84 (*p* < 0.05) and 3.40 (*p* < 0.05) respectively. The second risk factor associated with ECG changes was the cumulative dose of anthracyclines. Patients who were administered ≥ 350 mg/m^2^ of cumulative dose of anthracycline had a higher chance of obtaining ECG readings above the normal values compared to the patients who received a cumulative dose of anthracycline ˂350 mg/m^2^. The risk of changing the QTc interval was more than twice which was indicated by an OR of 2.14 (*p* < 0.05). However, the effect of the cumulative dose of anthracycline to increase the QRS duration above the normal value (OR; 2.00) was at the threshold *p-value* was 0.049.

**Table 7:** Analysis of the risk factors independently associated with cardiotoxicity (based on electrocardiography (ECG) analysis)

Characteristics	QRS duration				QTc interval		
	OR	95%CI	*p* value	OR		95%CI	*p* value
Age (Years) Reference; < 50 years							
50—59	1.43	0.73–2.83	0.299	1.70		0.75–3.82	0.201
60—79	2.84	1.39–5.80	0.004**	3.40		1.26–9.14	0.015*
BMI (kg/m^2^) Reference; normal							
Overweight	0.67	0.34–1.32	0.254	1.23		0.50–2.98	0.654
Obese	0.76	0.31–1.84	0.545	1.20		0.37–3.85	0.765
Nottingham grade of cancer							
Reference; Grade I							
Grade II	1.31	0.58–2.97	0.521	1.87		0.74–4.73	0.185
Grade III	1.39	0.58–3.33	0.458	1.87		0.82–6.50	0.111
Treatment with doxorubicin	1.07	0.45–2.54	0.876	0.79		0.38–1.64	0.526
Cumulative dose of anthracycline							
≥ 350 mg/m^2^	2.00	0.99–4.03	0.049*	2.14		1.16–3.96	0.014*
Diabetes mellitus	1.04	0.59–1.82	0.850	1.81		0.85–3.85	0.121
Dyslipidaemia	1.08	0.60–1.94	0.799	1.06		0.50–2.22	0.886
Trastuzumab	0.59	0.13–2.75	0.480	0.57		0.15–2.11	0.379
Chest wall irradiation	0.93	0.77–1.11	0.434	1.18		0.93–1.50	0.176

*OR* Odds ratio, *CI* Confidence interval, *BMI* Body Mass Index

*p* values *< 0.05, **< 0.01, ***< 0.001 considered as significant

### Biochemical analysis

The baseline mean NT-proBNP concentration of the patients recruited to the study was 91.25 pg/mL and cTnI was not detected in any of the patients at the baseline level. The comparison of mean concentrations of NT-proBNP and cTnI at four time points is shown in Table 8. The mean NT-pro BNP concentration one day after the first dose of anthracycline, one day after the last dose of chemotherapy, and six months after the completion of chemotherapy were 103.82 pg/ mL, 118.23 pg/ mL and 133.25 pg/ mL respectively and these values were significantly different (*p* < 0.001) from the baseline value. Although a considerable change in the concentration of cTnI was not detected in many patients one day after the first dose of anthracycline chemotherapy, some patients showed minimum detectable positive values where the mean concentration was 0.009 ng/ mL. However, the mean concentration of the cTnI was significantly elevated (*p* < 0.001) after the last dose and six months after the completion of anthracycline chemotherapy, and the mean values were 0.024 ng/mL and 0.040 ng/mL respectively. The reference value for the NT-proBNP was considered as ≤ 125.0 pg/ mL in ages between 0–74 years and the positive cTnI concentration was considered as ≥ 0.01 ng/ mL (minimum detectable level) and a significant elevation was considered as ≥ 0.04 ng/ mL [24–26]. There were 31.12% of patients with NT-proBNP concentration > 125.0 pg/ mL one day after the last dose of chemotherapy and 46.43% of patients six months after the completion of chemotherapy. There were 32.65% of patients who showed ≥ 0.01 ng/ mL of cTnI concentration one day after the first dose of chemotherapy. But none of them had a significant elevation of cTnI which indicates myocardial necrosis. There were 10.20% of patients with cTnI concentration ≥ 0.04 ng/ mL one day after the last dose of anthracycline chemotherapy and 41.33% of patients six months after the completion of chemotherapy.

**Table 8:** Comparison of biochemical parameters at four time points

Biochemical parameters Mean (95%CI)	One day prior to anthracycline chemotherapy (baseline)	One day after 1^st^ dose of anthracycline chemotherapy (FTP I)		One day after last dose of anthracycline chemotherapy (FTP II)	Six months after completion of anthracycline chemotherapy (FTP III)	
cTnI (ng/mL)	0.00	0.01 (0.007, 0.010)		0.02 (0.022, 0.025)	0.04 (0.039, 0.042)	
NT-pro BNP (pg/mL)	91.25 (89.5, 93.0)	103.82 (102.0, 105.6)		118.23 (116.5, 120.0)	133.25 (131.5, 135.0)	
Mean difference (95% CI)	FTP I—Baseline	FTP II—Baseline	FTP III—Baseline	FTP II—FTP I	FTP III—FTP I	FTP III—FTP II
cTnI (ng/mL)	0.01*** (0.00, 0.01)	0.02*** (0.02, 0.03)	0.04*** (0.03, 0.04)	0.01*** (0.01, 0.02)	0.03*** (0.02, 0.03)	0.02*** (0.01, 0.02)
NT-pro BNP (pg/mL)	12.6*** (9.3, 15.9)	27.0*** (23.7, 30.3)	42.0*** (38.7, 45.3)	14.4*** (11.1, 17.7)	29.4*** (26.2, 32.7)	15.0*** (11.7, 18.3)

All values are expressed as mean (95% CI) (n = 196). One-way Analysis of Variance (One-way ANOVA) test was applied to test the hypothesis of equal means at 0.05 level of significance. A p-vale of 0.000 + (* p* values *< 0.05, **< 0.01, ***< 0.001) indicated at least two different means. Then the Tukey’s multiple comparison test was applied to identify the different pairs of means)

*CI* confidence interval, *cTnI* cardiac troponin I, *NT-pro BNP* N terminal pro brain natriuretic peptide, *FTP* Follow up time point

FTP I; One day after 1st dose of anthracycline chemotherapy, FTP II; One day after last dose of anthracycline chemotherapy, FTP III; Six months after completion of anthracycline chemotherapy

Correlation analysis of biochemical changes with six continuous variables including age weight, height, BMI, cumulative dose of anthracycline, and chest wall irradiation is shown in Table 9. NT-proBNP concentration had a weak positive linear relationship with a cumulative dose of anthracycline (*r* = 0.311, *p* < 0.001) and chest wall irradiation (*r* = 0.238, *p* < 0.01). cTnI concentration six months after the completion of chemotherapy showed a positive linear relationship with all variables except the height of the patients. The cumulative dose of anthracycline had the strongest relationship (*r* = 0.659, *p* < 0.001) among these variables followed by chest wall irradiation, weight, BMI, and age.

**Table 9:** Correlation between the basic characteristics and the biochemical changes six months after the completion of anthracycline chemotherapy

Variables	Correlation coefficient (*p* value)	
	cTnI	NT-proBNP
Age	0.21 (0.003)**	0.12 (0.089)
Weight	0.33 (0.001)**	0.08 (0.259)
Height	0.08 (0.265)	0.02 (0.835)
BMI	0.32 (0.001)**	0.09 (0.228)
Cumulative dose of anthracycline	0.66 (0.002)**	0.31 (0.001)**
Radiation	0.41 (0.001)**	0.24 (0.001)**

*BMI* Body Mass Index, *cTnI* cardiac troponin I, *NT-proBNP* N-terminal pro brain natriuretic peptide

*p* values *< 0.05, **< 0.01, ***< 0.001 considered as significant

The Chi-Squared test was performed to investigate whether the biochemical changes are dependent on the changes in EF in echocardiography and ECG parameter changes six months after the completion of chemotherapy and results are shown in Tables 10, 11, 12, 13, 14, 15. There was a significant association (*p*˂0.01) between NT-proBNP concentration ˃125.00 ng/mL and EF changes ˃10% as well as ECG parameter changes six months after the completion of chemotherapy. cTnI concentration ˃0.04 ng/mL also had a significant association with the occurrence of sub-clinical cardiotoxicity and ECG parameter changes.

**Table 10:** Association of sub-clinical cardiotoxicity (EF changes ≥ 10%) with the cTnI changes six months after the completion of anthracycline chemotherapy

	cTnI < 0.04 ng/mL	cTnI ≥ 0.04 ng/mL
EF difference (EF_3_-EF_0_) < 10%	98 (74.2%)	34 (25.8%)
EF difference (EF_3_-EF_0_) ≥ 10%	17 (26.6%)	47 (73.4%)

*n* = 196, *p*-value = 0.001**

*cTnI* cardiac troponin I, *EF* Ejection fraction

*p* values *< 0.05, **< 0.01, ***< 0.001 considered as significant

**Table 11:** Association of QRS duration changes with the cTnI changes six months after the completion of anthracycline chemotherapy

	cTnI < 0.04 ng/mL	cTnI ≥ 0.04 ng/mL
QRS duration ≤ 120 ms	100 (64.9%)	54 (35.1%)
QRS duration > 120 ms	15 (35.7%)	27 (64.3%)

*n* = 196, *p*-value = 0.001**

*cTnI* cardiac troponin I

*p* values *< 0.05, **< 0.01, ***< 0.001 considered as significant

**Table 12:** Association of QTc interval changes with the cTnI changes six months after the completion of anthracycline chemotherapy

	cTnI < 0.04 ng/mL	cTnI ≥ 0.04 ng/mL
QTc interval ≤ 440 ms	91 (67.9%)	43 (32.1%)
QTc interval > 440 ms	24 (38.7%)	38 (61.3%)

*n* = 196, *p*-value = 0.002**

*cTnI* cardiac troponin I

*p* values *< 0.05, **< 0.01, ***< 0.001 considered as significant

**Table 13:** Association of sub-clinical cardiotoxicity (EF changes ≥ 10%) with the NT-pro BNP changes six months after the completion of anthracycline chemotherapy

	NT-pro BNP ≤ 125 pg/mL	NT-pro BNP > 125 pg/mL
EF difference (EF_3_-EF_0_) < 10%	80 (60.6%)	52 (39.4%)
EF difference (EF_3_-EF_0_) ≥ 10%	25 (39.1%)	39 (60.9%)

*n* = 196, *p*-value = 0.005**

*EF* Ejection fraction, *NT-proBNP* N-terminal pro brain natriuretic peptide

*p* values *< 0.05, **< 0.01, ***< 0.001 considered as significant

**Table 14:** Association of QRS duration changes with the NT-pro BNP changes six months after the completion of anthracycline chemotherapy

	NT-pro BNP ≤ 125 pg/mL	NT-pro BNP > 125 pg/mL
QRS duration ≤ 120 ms	92 (59.7%)	62 (40.3%)
QRS duration > 120 ms	13 (31.0)	29 (69.0%)

*n* = 196, *p*-value = 0.001**

*NT-proBNP* N-terminal pro brain natriuretic peptide

*p* values *< 0.05, **< 0.01, ***< 0.001 considered as significant

**Table 15:** Association of QTc interval changes with the NT-pro BNP changes six months after the completion of anthracycline chemotherapy

	NT-pro BNP ≤ 125 pg/mL	NT-pro BNP > 125 pg/mL
QTc interval ≤ 440 ms	85 (63.4%)	49 (36.6%)
QTc interval > 440 ms	20 (32.3%)	42 (67.7%)

*n* = 196, *p*-value = 0.000***

*NT-proBNP* N-terminal pro brain natriuretic peptide

*p* values *< 0.05, **< 0.01, ***< 0.001 considered as significant

Binary logistic regression analysis was performed to assess the risk factors associated with biochemical changes of the patients recruited to the study and results are shown in Table 16. According to the results, the only risk factor associated with high NT-proBNP was the cumulative dose of anthracycline. Patients receiving a cumulative dose of anthracycline ≥ 350 mg/m^2^ showed a significant (*p* < 0.05) association with the occurrence of cardiotoxicity with NT-proBNP concentration > 125 pg/mL. It was about two times greater than the patients receiving < 350 mg/m^2^ of anthracyclines. Although the other risk factors including age ≥ 60 years, overweight, obesity, diabetes mellitus, dyslipidemia and chest wall irradiation had more than one OR, they were not significant. However, many risk factors showed a significant association with the occurrence of cardiotoxicity by means of an elevated cTnI concentration in blood. Chest wall irradiation had a significant effect (*p* < 0.05) on the elevation of cTnI concentration. It showed an OR of 12.93 (CI; 1.68—99.60) which indicated that the patients who undergo chest wall irradiation have 12 times more risk of having an elevated concentration of cTnI than the patients without any chest wall irradiation. The cumulative dose of anthracycline ≥ 350 mg/m^2^ (0R; 7.27, *p* < 0.001) followed by obesity (OR; 4.06, *p* < 0.01) and overweight (OR; 2.60, *p* < 0.01) also had significant associations with the occurrence of cardiotoxicity by means of elevation in cTnI. Patients with Nottingham grade III cancer (OR; 2.70) were also identified as having a significant (*p* < 0.05) risk factor in the event of elevated cTnI than those having grade I cancer in the present study.

**Table 16:** Analysis of the risk factors independently associated with cardiotoxicity (based on biochemical analysis)

Characteristics	cTnI			NT-proBNP		
	OR	95%CI	*p* value	OR	95%CI	*p* value
Age (Years) Reference; Age < 50 years						
50—59	0.55	0.27—1.11	0.095	0.70	0.35—1.38	0.302
60—79	1.46	0.73—2.92	0.280	1.32	0.66—2.64	0.425
BMI (kg/m^2^) Reference; Normal						
Overweight	2.60	1.34—5.04	0.005**	1.19	0.63—2.28	0.593
Obese	4.06	1.76—9.40	0.001**	2.04	0.93—4.51	0.077
Nottingham grade of cancer						
Reference; Grade I						
Grade II	1.06	0.45—2.52	0.885	1.27	0.56—2.90	0.566
Grade III	2.88	1.17—7.09	0.022*	1.79	0.75—4.30	0.190
Cumulative dose of anthracycline ≥ 350 mg/m^2^	7.28	3.81—13.84	0.001**	2.09	1.18—3.70	0.014*
Treatment with doxorubicin	1.16	0.57—2.38	0.685	0.78	0.39—1.58	0.498
Diabetes mellitus	1.02	0.58—1.82	0.933	1.09	0.62—1.92	0.772
Dyslipidaemia	0.79	0.43—1.44	0.445	1.00	0.55—1.79	0.991
Trastuzumab	0.77	0.25—2.40	0.659	0.86	0.29—2.57	0.781
Radiation	12.93	1.68—99.60	0.014*	3.07	0.96—9.79	0.057

*OR* Odds ratio, *CI* Confidence interval, *BMI* Body Mass Index, *cTnI* cardiac troponin I, *NT-proBNP* N-terminal pro brain natriuretic peptide

*p* values * ˂ 0.05, **˂ 0.01, *** ˂0.001 considered as significant

## Discussion

Dose-dependent cardiotoxicity of anthracyclines is considered the most serious complication of anthracycline therapy and it has a negative impact on the cardiac outcome of cancer patients and a major limitation on their therapeutic potential [27]. As the prognosis of cancer patients is very poor due to chemotherapy-induced cardiotoxicity, early diagnosis is important to prevent the fatal outcomes associated with anthracycline-induced cardiotoxicity as well as to reduce the development of late chronic cardiotoxicities [28]. However, in Sri Lanka, studies have not been reported on anthracycline-induced cardiotoxicity in cancer patients other than a study performed by the same authors based on an echocardiographic analysis [22]. Therefore, the current study was conducted to detect the prevalence of anthracycline-induced cardiotoxicity in adult breast cancer patients who were admitted to the oncology unit at the Teaching hospital, Karapitiya, Galle, Sri Lanka based on ECG parameters and a biochemical analysis.

In anthracycline-induced cardiotoxicity, the incidence of clinical decompensation is about 2–4% and sub-clinical structural changes are about 9–11% while biomarker rise can be seen in about 30–35% of patients [1]. As sub-clinical cardiotoxicity is more prevalent in patients on anthracycline chemotherapy, early detection may increase the survival rate of cancer patients [29]. For the detection of anthracycline-induced acute and early-onset chronic cardiotoxicity, the present study used two detection methods including ECG and cardiac biomarkers such as cTnI and NT-proBNP. The ECG measurements help to identify anthracycline-induced acute cardiac changes such as prolongation of QRS duration and QTc interval, but they may not be specific and therefore, the imaging techniques are recommended [30]. Although echocardiography is a commonly used traditional non-invasive and non-radioactive technique which can be used in daily practice to detect cardiotoxicity, it is not a suitable method to detect cardiotoxicity at a very early stage as it detects the abnormality after having some degree of damage to the myocardium [31]. Troponins are highly sensitive and specific cardiac biomarkers located in the myocytes and they are released into the circulation when there is a myocardial injury therefore, they are considered good markers to detect and monitor cardiac injury [31, 32]. NT-proBNP is identified as a reliable marker to investigate anthracycline-induced cardiotoxicity as it correlates with left ventricular pressure and volume changes and myocardial tissue damage and it has a significant relationship with both systolic and diastolic dysfunction of the heart [32]. Furthermore, BNP has a high negative predictive value (98%) which can rule out ventricular dysfunction when there is a low level of BNP in plasma and this marker can be used to identify both acute and chronic cardiotoxicity.

Echocardiography is a convenient diagnostic tool to identify both clinical and sub-clinical cardiotoxicity. LVEF measurement taken by echocardiography is commonly used to assess cardiac systolic functions and it is also widely used in the prognosis of cardiac disease [33]. The previous study conducted by the same authors found sub-clinical cardiotoxicity in 33% of patients six months after the completion of anthracycline chemotherapy based on the echocardiographic analysis [22] and the results were consistent with previous studies [34, 35].

ECG is a widely used low-cost method to detect chemotherapy-induced cardiotoxicity in oncology [17]. It is reported that 20–30% of patients who have sub-acute and acute cardiotoxicity may have transient ECG abnormalities such as QRS voltage reduction, QTc interval prolongation and ST and T wave flattening [13]. In the present study, measurements of the PR interval, QRS duration and QTc interval were used in the detection of acute and chronic cardiotoxicities as the duration of these parameters are widely recognized in the diagnosis and monitoring of heart diseases and they have been used in previous studies related to anthracycline-induced cardiotoxicity [13, 17, 36, 37]. In the present study, QRS duration and QTc interval showed changes in the acute phase as well as in the chronic phase and PR interval didn’t show much variation with the anthracycline treatment. The reason for this may be due to the higher damage caused by anthracycline-induced cardiotoxicity to the left ventricles. Therefore, patients are more prone to left ventricular dysfunction [2]. In the present study, a less percentage of patients showed acute cardiotoxicity while > 30% of patients showed early-onset chronic progressive cardiotoxicity utilizing QRS and QTc interval and these results were in line with the results of the study done by Horacek, et al. [17].

Another study done in Uganda on adult patients who underwent anthracycline chemotherapy showed a significant (p < 0.05) increase in QTc interval and a decrease in QRS duration three months after the completion of anthracycline chemotherapy [13]. Although the absolute values are not the same, both studies showed apparent changes in QTc interval in early-onset chronic cardiotoxicity induced by anthracyclines. Although in the Ugandan study, there is a decline in QRS duration after anthracycline chemotherapy, several studies including animal and human studies have shown that the duration of QRS becomes prolonged after anthracycline chemotherapy [38–42].

In the present study correlation between ECG parameter changes and basic biological factors including age, height, weight, BMI and clinical factors such as cumulative dose of anthracycline and radiation in early-onset chronic cardiotoxicity were investigated and a correlation was observed only with age and the cumulative dose of anthracyclines. However, many previous studies have shown that cardiomyopathy induced in anthracycline chemotherapy may be correlated with many factors including age, gender, BMI, anthracycline dose and radiation therapy [43–46]. Lipshultz, et al. have apparently shown that a cumulative dose of anthracycline has a considerable relationship with the prolongation of QTc interval as anthracyclines cause cardiac arrhythmia [46]. One of his previous studies has shown that late cardiotoxicity is common in the female sex and in children who were subjected to high doses of anthracycline chemotherapy in childhood cancer [1]. When investigating the risk factors associated with ECG parameters above the reference values, the cumulative dose of anthracycline was the most associated risk factor. A study done by Desai, et al. on electrocardiograms for cardiomyopathy risk stratification in children with anthracycline exposure has also shown that the cumulative dose of anthracycline is a significant predictor of an increase in QTc interval [16].

As the sub-clinical cardiotoxicity was evident six months after the completion of chemotherapy, the association of ECG abnormalities with > 10% deviation of EF compared to the baseline EF in echocardiography was investigated and a significant association between the two diagnostic methods was observed. A study done by Horacek, et al. have also shown that QRS voltage lowering and QTc interval prolongation have significant correlations with the left ventricular dysfunction identified by echocardiography [17]. Several other studies have also used both techniques for the detection of late cardiotoxicity in patients treated with anthracyclines [13, 30, 47, 48].

More NT-proBNP is released when there is an elevation in wall stress and filling pressure in the left ventricles. Many previous studies have shown that anthracycline-induced cardiotoxicity increases ventricular dysfunction and in turn increases the release of BNP [49–55]. cTnI is normally absent in circulation and is released when there is cardiomyocyte damage due to inflammation, ischaemia, apoptosis or oxidative stress [56] and several previous studies have shown the involvement of these mechanisms in anthracycline-induced cardiotoxicity which increases the cTnI concentration in blood [27, 54, 57–61]. Therefore, these two markers are considered the most sensitive and specific markers to detect the prevalence of anthracycline-induced cardiotoxicity in cancer patients.

In the present study, there were > 30% of patients with increased cTnI and NT-proBNP concentrations one day after the first dose of chemotherapy compared to the baseline values. However, those changes were not in the cardiotoxic range and these kinds of transient changes have been revealed in some previous studies as well [62, 63].

According to our results, cardiotoxic evidence was observed at the second and third follow-up time points of anthracycline chemotherapy. A study done by Sawaya, et al. have shown that about 32% of patients in their cohort study who were followed up to 15 months using biochemical and imaging studies were diagnosed with cardiotoxicity [61]. Another follow-up study conducted for four to seven months by Cardinale et al. showed that a cTnI > 0.04 ng/mL was detected in 53% of patients after the completion of chemotherapy. A study done on peadiatric patients regarding changes in biomarkers during nine months of doxorubicin treatment showed that cTnI concentration increased in 47% of patients after the treatment and NT-proBNP level increased in 48% of patients after the treatment.

According to the present study, a significant elevation of cTnI was correlated with age, weight, BMI, cumulative dose of anthracycline and chest wall irradiation and NT-proBNP has a significant correlation with the cumulative dose of anthracyclines and chest wall irradiation. However, univariate binary logistic regression revealed that overweight, obesity, cumulative dose of anthracycline > 350 mg/m^2^ and chest wall irradiation are the risk factors for the abnormal cTnI while only the cumulative dose of anthracycline > 350 mg/m^2^ is a risk factor for abnormal NT-proBNP. In the multivariate analysis, only the age of ˃60 years and cumulative dose of ˃350 mg/m^2^ were identified as risk factors for the changes in cTnI concentrations. Consistent with our results, in previous studies, cumulative dose of anthracycline, female sex, body weight, age ˃65 years, treatment with radiation, cyclophosphamide, taxanes, genetic factors, pre-existing cardiac diseases have been identified as risk factors [64–67]. Several other studies also showed that there is a significant association between the cumulative dose of anthracycline and the increase in biomarkers including cTnI and NT-proBNP [1, 68–70]. Chest wall irradiation was another risk factor most associated with elevated levels of cTnI and it showed 12 times more risk than non-irradiated patients. A study done by Darby, et al. showed that there is a higher risk of having ischaemic heart diseases following radiotherapy and even an increase in 1 Gy beyond the average radiation therapy (0.03 – 27.72) can increase the cardiac events by 7.4%, especially with left side chest wall irradiation [71]. Several other studies also have proven this effect [72, 73]. Patients who were positive for Her-2 receptors were treated with trastuzumab after the anthracycline chemotherapy. Some previous studies reported that trastuzumab induce cardiac dysfunction in patients and this may be further exaggerated if the trastuzumab is administered after the anthracycline therapy [74–76]. However, these studies suggested that it takes a longer time of duration to induce significant cardiotoxicity. Therefore, in the present study, it was suggested that the contribution of trastuzumab for sub-clinical cardiotoxicity may be minimum as only a minor percentage of patients (about 7%) received the trastuzumab and it was prescribed as the low-dose long-term therapy.

In the present study, the Chi-Squared test revealed that there is a strong association between LVEF and the changes in cTnI concentration than the NT-proBNP concentration six months after the completion of chemotherapy. The reason for this observation would be that NT-proBNP is usually increased with the volume or pressure overload, but LVEF changes and release of cTnI require structural damage to the cardiomyocytes [62, 77]. This finding was in line with many studies which reported a reduction of LVEF (sub-clinical cardiotoxicity) that is strongly associated with an increased cTnI but less associated with NT-proBNP concentration [57, 59, 78–82].

In the present study, patients who are administering with anthracycline and cyclophosphamide were recruited since there were no patients who are prescribed only with anthracycline drugs in the actual clinical set up and it is always used combination with other drugs. However, this was the only combination which include only two drugs. Therefore, there may be a contribution to induce sub-clinical cardiotoxicity from the cyclophosphamide as well which cannot be controlled by the investigators. This is one of the limitations of this study, since previous reports mentioned that cyclophosphamide induced cardiotoxicity is a well-known adverse effect [83, 84]. According to the Dhesi et. al., incidence of acute heart failure may be between 7 to 33% when the patient is administered with > 150 mg/kg of cyclophosphamide [83]. However, there are no strong evidences regarding this cardiotoxicity since this drug is always used with other cardiotoxic drugs and reports available are based on the case reports and different case reports mentioned different doses and different percentages of incidences [83, 84].

In the present study, the prevalence of sub-clinical cardiac dysfunction was higher (33%) compared to other Asian studies [34, 35]. The reason for this may be due to the higher rate of risk factors which cannot be excluded from this study. However, overt clinical symptoms couldn’t be seen in these patients. Nowadays in other countries, pegylated liposomal doxorubicin provides tumour-targeted efficacy without many of the toxicities associated with conventional doxorubicin, including myelosuppression, alopecia, nausea and vomiting, and most importantly, cardiac toxicity [85]. However, in developing countries including Sri Lanka this is not affordable and therefore, the results of this study provide scientific evidence that cancer patients on anthracycline chemotherapy must be screened for cardiotoxicity periodically after the initiation and completion of anthracycline chemotherapy to minimize any future occurrence of anthracycline-induced cardiotoxicity to ensure the increased quality of life in cancer survivors.

There were several other limitations of this study which need to be mentioned. This study was conducted in a single tertiary care center, therefore, data are more related to the Southern province of Sri Lanka. Only the female patients with breast cancer could be selected for the study to maintain the consistency and to reduce the interference from multiple other factors related to anthracycline induced cardiotoxicity. During the six months follow-up period, patients were given second line treatments as well. They may also have exposed to mediastinal irradiation. Therefore, the final ECG and biochemical data obtained may be contaminated by the administration of non-anthracycline drugs and their toxicities and by the radiation therapy.

## Conclusion

According to the biochemical analysis of the present study, > 40% of patients showed early onset chronic cardiotoxic changes six months after the completion of anthracycline chemotherapy. However, ECG changes were more noticeable one day after the first dose of anthracycline chemotherapy. Since these results confirmed the unavoidable cardiotoxic changes following anthracycline chemotherapy, it is recommended to carry out long-term follow-ups in all patients who were treated with anthracycline therapy to increase their quality of life as cancer survivors.

## Data Availability

The datasets used and/or analyzed during the current study are available from the corresponding author upon reasonable request.

## Abbreviations

AC: Anthracycline and cyclophosphamide
BP: Blood pressure
BMI: Body mass index
cTnI: Cardiac troponin I
ECG: Electrocardiography
ELISA: Enzyme linked immunosorbent assay
ER: Oestrogen receptor
FTP: Follow up time point
Her 2: Human epidermal growth factor receptor 2
LVEF: Left ventricular ejection fraction
NT-pro BNP: N terminal pro brain natriuretic peptide
NADPH: Nicotinamide adenine dinucleotide phosphate
PR: Progesterone receptor
ROS: Reactive oxygen species
